# *fastQ_brew*: module for analysis, preprocessing, and reformatting of FASTQ sequence data

**DOI:** 10.1186/s13104-017-2616-7

**Published:** 2017-07-12

**Authors:** Damien M. O’Halloran

**Affiliations:** 1 Institute for Neuroscience, The George Washington University, 636 Ross Hall, 2300 I St. N.W., Washington, DC 20052 USA; 2 Department of Biological Sciences, The George Washington University, 636 Ross Hall, 2300 I St. N.W., Washington, DC 20052 USA

**Keywords:** FASTQ, NGS, Sequencing

## Abstract

**Background:**

Next generation sequencing datasets are stored as FASTQ formatted files. In order to avoid downstream artefacts, it is critical to implement a robust preprocessing protocol of the FASTQ sequence in order to determine the integrity and quality of the data.

**Results:**

Here I describe *fastQ_brew* which is a package that provides a suite of methods to evaluate sequence data in FASTQ format and efficiently implements a variety of manipulations to filter sequence data by size, quality and/or sequence. *fastQ_brew* allows for mismatch searches to adapter sequences, left and right end trimming, removal of duplicate reads, as well as reads containing non-designated bases. *fastQ_brew* also returns summary statistics on the unfiltered and filtered FASTQ data, and offers FASTQ to FASTA conversion as well as FASTQ reverse complement and DNA to RNA manipulations.

**Conclusions:**

*fastQ_brew* is open source and freely available to all users at the following webpage: https://github.com/dohalloran/fastQ_brew.

## Background

FASTQ format has become the principal protocol for the exchange of DNA sequencing files [1]. The format is composed of both a nucleotide sequence as well as an ASCII character encoded quality score for each nucleotide. Each entry is four lines, with the first line starting with a ‘@’ character followed by an identifier. The second line is the nucleotide sequence. The third line starts with a ‘+’ character and optionally followed by the same sequence identifier that was used on the first line. The fourth line lists the quality scores for each nucleotide in the second line. In order to evaluate the quality of the FASTQ dataset and to avoid downstream artefacts, it is imperative for the user to employ robust quality control and preprocessing steps prior to downstream FASTQ applications. Furthermore, FASTQ has now become widely used in additional downstream applications and pipelines, and so diverse preprocessing tools are necessary to handle various FASTQ file manipulations [2, 3]. Here, I describe *fastQ_brew*, which is a robust package that performs quality control, reformatting, filtering, and trimming of FASTQ formatted sequence datasets.

## Implementation

*fastQ_brew* was developed using Perl and successfully tested on Microsoft Windows 7 Enterprise ver.6.1, Linux Ubuntu 64-bit ver.16.04 LTS, and Linux Mint 18.1 Serena. *fastQ_brew* does not rely on any dependencies that are not currently part of the Perl Core Modules (http://perldoc.perl.org/index-modules-A.html), which makes *fastQ_brew* very straight forward to implement. *fastQ_brew* is composed of two separate packages: *fastQ_brew.pm* and *fastQ_brew_Utilities.pm*. *fastQ_brew_Utilities.pm* provides *fastQ_brew.pm* with access to various subroutines that are called to handle FASTQ manipulations and quality control. The *fastQ_brew* object is instantiated by calling the constructor subroutine called “new” which creates a ‘blessed’ object that begins gathering methods and properties by calling the *load_fastQ_brew* method. Once the object has been populated, the user can call *run_fastQ_brew* to begin processing the FASTQ data. Sample data are provided at the GitHub repo and directions for usage are described in the README.md file.

The command-line arguments supplied to the *fastQ_brew* object are as follows: (1) -*lib*, which can be either *sanger* or *illumina*; (2) -*path*, specifies the path to the input file (can use “./” for current directory with UNIX or “.\” on Windows cmd); (3) -*i*, this is the name of the file containing the FASTQ reads; (4) -*smry*, return summary statistics table on the unfiltered data and filtered data; (5) -*qf*, this option will filter reads by Phred (also called Q score) quality score—any reads having an average Phred score below the threshold will be removed: e.g. -*qf* = 20 will remove reads with Phred scores below 20; (6) -*lf*, this will filter reads below a specified length; (7) -*trim_l*, will trim the specified number of bases from the left end of each read; (8) -*trim_r*, same as left-trim except that here the reads will be trimmed from the right side; (9) -*adpt_l*, will remove a specified adapter sequence from the left end of a read; (10) -*adpt_r*, same as -*adpt_l* except that here the reads will be trimmed from the right side; (11) -*mis_l*, allows for a specified number of mismatches between the user provided -*adpt_l* sequence and each read e.g. a mismatch = 1, would match a hypothetical 3 base adapter, TAG, to the left end of a sequence that started with TAG or AAG or TAA or any of the nine possibilities; (12) -*mis_r*, same as -*mis_l* except that this relates to the *adpt_r* sequence supplied by the user; (13) -*dup*, removes duplicate reads; (14) -*no_n*, removes reads that contain non-designated bases i.e. bases that are not A, G, C or T e.g. N; (15) -*fasta*, this option will convert the FASTQ file to FASTA format; (16) -*rev_comp*, will reverse complement reads in the supplied FASTQ file; (17) -*rna*, will convert each read to the corresponding RNA sequence in the supplied FASTQ file; (18) -*clean*, option to delete temporary files created during the run. If the *summary* option is selected, *fastQ_brew* will return a results table to STDOUT with summary statistics of the FASTQ data file prior to filtering and after filtering. The summary report will provide a table detailing max, min, and average GC% values for all reads; max, min, and average read lengths, max, min, and average Phred scores, and max, min, and average error probabilities. The Phred score (denoted as Q) represents the probability of an error for each base, and is logarithmically related to the base-calling error probability, *P* such that:

$$Q = - 10\log_{10} P$$

or

$$P = 10\frac{ - Q}{10}$$

In the case of arguments 15–17 above, a new file will be generated in each case, whereas for all other options the user-supplied arguments will be chained together to return a single filtered file.

## Results

**Fig. 1:**
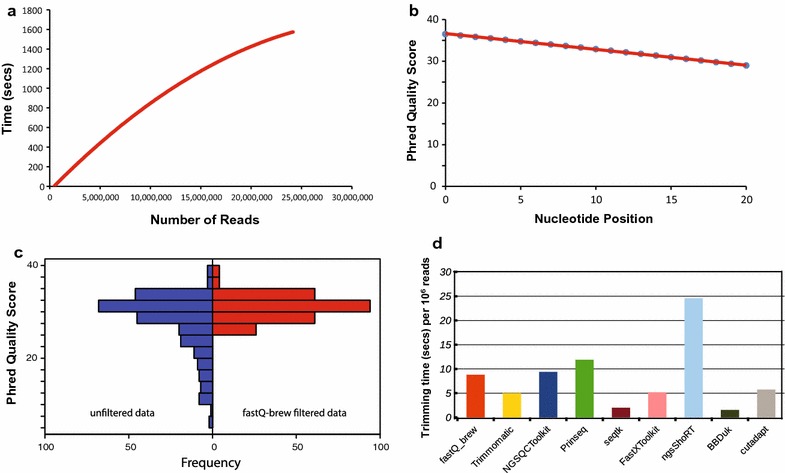
**a** Performance testing of *fastQ_brew*. FASTQ formatted files containing different numbers of reads (110 MB [462,664 reads] to 4.5 GB [24,159,698 reads]) were provided as input to *fastQ_brew* and ran using default settings to return summary statistics for each dataset. **b** Relationship between nucleotide position and Phred quality score. *fastQ_brew* was used to determine the average Phred quality score from a FASTQ dataset comprising 462,664 reads after the length trimming methods was invoked to trim each read from position 1–20. A negative correlation between increasing nucleotide position and quality was observed. **c** The quality filter method within *fastQ_brew* was tested by plotting the Phred scores before (*blue bars*) and after (*red bars*) quality filtering. After filtering, there was a shift in the distribution of reads towards higher quality Phred values. **d** Execution speed for commonly used FASTQ filtering tools were compared with *fastQ_brew*. For all analyses, the same file and trimming task was applied. The following software were compared and presented: *fastq_brew ver 1.0.2, trimmomatic ver 0.36, NGSQCToolkit ver 2.3.3, Prinseq ver 0.20.4, seqtk, Fastxtoolkit ver 0.0.13, ngsShoRT ver 2.2*, *BBDuk ver 37.22*, and *Cutadapt ver 1.9.1*

Testing of *fastQ_brew* was performed by plotting runtime against file size (Fig. 1a). FASTQ formatted sequence data from 110 MB (462,664 reads) to 4.5 GB (24,159,698 reads) in size were used to benchmark the runtime of *fastQ_brew*. In each case, *fastQ_brew* efficiently returned summary statistics from each file in 36 s for 110 MB FASTQ file to 25 min and 33 s for 4.5 GB. The runtime will scale with the number of methods called within *fastQ_brew*.

To evaluate more specific methods within *fastQ_brew*, the relationship between nucleotide position within a given read and the corresponding Phred quality score was determined (Fig. 1b). This method tested the trimming and Phred calculation methods within *fastQ_brew*. The Phred quality score is used as a metric to determine the quality of a given nucleotide’s identification within a read [4]. Phred quality scores are related (logarithmically) to the base-calling error probabilities [5] (see equation above). The average Phred quality scores for a randomly chosen FASTQ data file after left-side trimming (-*trim_l*) method invocations within *fastQ_brew* from position 1–20 were plotted (Fig. 1b). There was a negative correlation between increasing nucleotide position and Phred quality score (*R* ^*2*^ = −0.99969), that is, bases closer to the beginning of each read exhibit higher Phred quality scores, as compared with nucleotides closer to the middle of the read. This observation is in keeping with previous observations on Phred quality across reads [6–8] (http://www.bioinformatics.babraham.ac.uk/projects/fastqc/). The data set used in this test was comprised of 462,664 reads with an average read length of 99 bases. The smallest read length was 25 bases and the largest was 100 bases.

To further examine the quality filtering method of *fastQ_brew*, FASTQ data were downloaded from the NCBI sequence read archive (SRA—https://www.ncbi.nlm.nih.gov/sra) using the *sra*-*toolkit* (https://github.com/ncbi/sra-tools). Distribution of read quality was plotted prior to filtering (blue bars) and after filtering (red bars) using *fastQ_brew* revealing a shift in Phred scores towards increased quality after filtering (Fig. 1c).

Finally, to compare *fastQ_brew* to other FASTQ filtering tools, I examined the execution time for some of the most commonly used filtering tools in trimming FASTQ data, and compared their execution speeds to that of *fastQ_brew*. For all analyses, the same FASTQ file was used, and in each case methods were invoked to trim 8 bases from the left and right sides of every read in the file. The following software were used: *fastq_brew ver 1.0.2; Trimmomatic ver 0.36* [9]*; NGSQCToolkit ver 2.3.3* [6]*; Prinseq ver 0.20.4* [10]; *seqtk* (https://github.com/lh3/seqtk); *Fastxtoolkit ver 0.0.13* (http://hannonlab.cshl.edu/fastx_toolkit/index.html); *BBDuk ver 37.22* (http://jgi.doe.gov/data-and-tools/bbtools/bb-tools-user-guide/bbmap-guide/); *ngsShoRT ver 2.2* [11]; and *Cutadapt ver 1.9.1* (http://journal.embnet.org/index.php/embnetjournal/article/view/200). For some other software tools, this exact invocation was not possible due to limitations on the trimming method. The data from this analysis is presented in Fig. 1d. *fastQ_brew* compares well with other commonly employed filtering tools. The fastest tool was *BBDuk* which finished trimming all reads in only 1.532 s, and this was followed very closely by *seqtk* which completed the task in 1.99 s. By examining across these tools we can obtain some insight into how the execution speeds for *fastQ_brew* compares with commonly used trimming software. However, it is important to point out that each tool offers many specific adaptations and features that are not reflected in a basic trimming task, and while speed is important when dealing with very large data-sets, other features that include accessibility, documentation, ease of use, as well as applicability of options are equally important.

In summary, I here describe *fastQ_brew*, a very lightweight Perl package for robust analysis, preprocessing, and manipulation of FASTQ sequence data files. The main advantage of *fastQ_brew* is its ease of use, as the software does not rely on any modules that are not currently contained within the Perl Core. *fastQ_brew* is freely available on GitHub at: https://github.com/dohalloran/fastQ_brew.
